# Coinfection of Malaria and Bacterial Pathogens among Acute Febrile Patients in Selected Clinics in Ghana

**DOI:** 10.4269/ajtmh.23-0099

**Published:** 2023-09-25

**Authors:** Janice N. A. Tagoe, Clara Yeboah, Eric Behene, Selassie Kumordjie, Shirley Nimo-Paintsil, Naiki Attram, Edward O. Nyarko, John Ayite Carroll, Anne T. Fox, Chaselynn Watters, Kwadwo Koram, Abraham Kwabena Anang, Terrel Sanders, Andrew G. Letizia

**Affiliations:** ^1^ Noguchi Memorial Institute for Medical Research, Accra, Ghana;; ^2^ U.S. Naval Medical Research Unit-No.3, Ghana Detachment, Accra, Ghana;; ^3^ 37 Military Hospital, Accra, Ghana;; ^4^ 2 Garrison Clinics, Sekondi/Takoradi, Ghana;; ^5^ U.S. Naval Medical Research Unit-2 Detachment, Singapore

## Abstract

Malaria remains the leading cause of acute febrile illness (AFI) in Africa despite successful control measures and programs. Acute febrile illnesses can be misdiagnosed as malaria as a result of the overlapping spectrum of nonspecific symptoms or may not be pursued because of limited diagnostic capabilities. This study investigated potential etiologies of AFIs in Ghana and determined the relationship between coinfection between malaria and Q fever, leptospirosis, and culturable bacteria in febrile patients. Participants were enrolled between July 2015 and December 2019 from four Ghanaian military treatment facilities. Of the 399 febrile participants, 222 (55.6%) males and 177 (44.6%) females were enrolled. Malaria was diagnosed in 275 (68.9%) participants. Malaria coinfection occurred with leptospirosis, Q fever, and blood-cultured bacteria in 11/206 (5.3%), 24/206 (11.7%), and 6/164 (3.7%) participants, respectively. Among the 124 malaria-negative samples, the positivity rates were 4.1% (3/74), 8.1% (6/74), and 3.6% (2/56) for leptospirosis, Q fever, and bacterial pathogens isolated from blood culture, respectively. The majority of documented clinical signs and symptoms were not significantly associated with specific diseases. Approximately 10% of malaria-positive participants also had evidence suggesting the presence of a bacterial coinfection. Therefore, even in the case of a positive malaria test, other pathogens contributing to febrile illness should be considered. Understanding the frequency of malaria coinfection and other etiological agents responsible for AFIs will improve diagnosis and treatment and better inform public health knowledge gaps in Ghana.

## INTRODUCTION

Malaria often presents with nonspecific symptoms and can be difficult to distinguish clinically owing to limited diagnostic capabilities and resources in low- to middle-income countries.^1^ Unfortunately, the overdiagnosis of malaria and the underdiagnosis of other etiologies may be attributed to the similarities between nonspecific acute febrile illness (AFI) syndromes.^2^

Improved control measures have significantly decreased the worldwide incidence of malaria,^3^ and in 2010 the WHO recommended antimalarial treatment of AFI only after parasitological confirmation.^4^ However, in the African sub-region, malaria remains the leading diagnosis given to patients with fever^5^ because the true etiology of fever is often difficult to ascertain in these resource-limited regions. In Ghana, which is considered a high malaria transmission area, there is little epidemiologic data regarding the possible causes of non-malarial fevers. Once malaria is suspected, clinicians typically do not pursue other potential etiologies, even in circumstances where coinfections could be present. Interestingly, one study addressing the underestimation of non-malaria fevers reported that about 60% of febrile illnesses were caused by different pathogens, even in highly endemic malarial zones.^6^ Another recent study found that 5% of children under the age of 5 years have bacterial bloodstream infections, caused most frequently by non-typhoidal *Salmonella* and *Salmonella typhi*.^7^

Bacterial diseases such as Q fever and leptospirosis are known to cause AFI syndromes and have been reported in humans, livestock, and rodents in Ghana,^8^^–^^10^ but they have not been well studied in Ghana. Although these bacteria and reservoirs are present in Ghana, these pathogens require testing not typically available at medical treatment facilities and could represent a portion of undiagnosed AFIs. Understanding the prevalence of malaria coinfection and other possible causes of febrile infections should allow healthcare providers to improve diagnostic certainty and guide treatment regimens for their patients,^11^ especially considering that coinfections often manifest with greater severity and higher mortality.^8^ Therefore, this study aimed to identify the potential zoonotic or bacterial etiologies of AFIs in Ghana and determine their coinfections with malaria.

## MATERIALS AND METHODS

### Study design.

This was a cross-sectional study that enrolled febrile patients from inpatient and outpatient settings in Ghana. The study was conducted from July 2015 to December 2019 at the 37 Military Hospital located in Accra and three military clinics in Sekondi-Takoradi. The clinics included the Western Naval Command Medical Center in Sekondi, as well as the 2 Medical Reception Station (2MRS Armed Forces clinic) and the Air Force Medical Center in Takoradi. These clinics in the Western Region of Ghana serve primarily the military and their families, but also civilians.

### Recruitment of study participants.

Patients between 1 month and 65 years old presenting with a documented or self-reported fever (≥ 38°C or history of fever within the last 24 hours) without a localizing etiology (i.e., otitis media, meningitis, diarrheal syndromes) were eligible for enrollment. All patients with AFIs seeking medical care at the medical centers were screened for eligibility to participate in the study. The informed consent process was completed for all eligible individuals. For participants less than 18 years old, both child assent and parental permission were obtained. Questionnaires were administered to capture demographic and clinical data.

### Ethical considerations.

Ethical approval was obtained from the institutional review boards of the Ghana Health Service (GHS-ERC 14/05/13), Ghana Armed Forces (GAF-IRB 002/13), Noguchi Memorial Institute for Medical Research (NMIMR-IRB 126/12-13), and the U.S. Naval Medical Research Center (NMRC-IRB NAMRU3.2014.0003). The study protocol was approved by the aforementioned institutional review board in compliance with all applicable federal regulations governing the protection of human subjects.

### Sample collection and processing.

**Figure 1.**
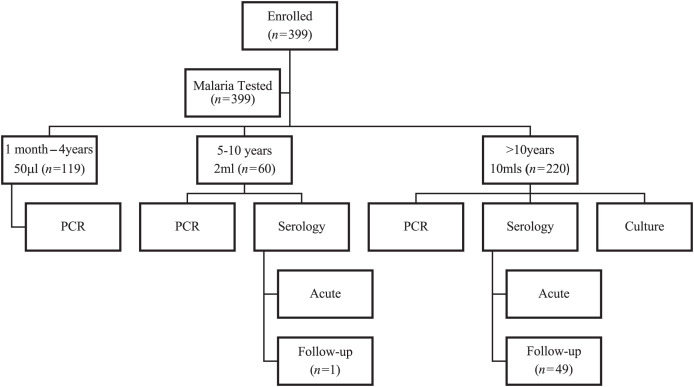
A flowchart of the study evaluating 399 acute febrile patients with respect to age. This figure shows the workflow for the number of participants sampled for the different tests based on their age group. PCR = polymerase chain reaction.

Blood samples were collected via needle prick (1 month to 4 years) or venipuncture (5 years and above) from all participants. The volume of blood collected was age dependent but did not exceed 10 mL. At the study sites, a specimen of 1 to 10 mL of blood was collected from study participants based on age group (Figure 1) and put in different collection tubes/bottles based on the test to be performed. About 2 to 5 mL of blood was put in a sera separation tube, and samples were centrifuged at 1,500 × *g* for 15 minutes to obtain sera. One to three milliliters was placed in the BACTEC bottles for bacteria culture, along with two to three drops (approximately 60 μL) of EMJH medium for leptospiral culture. At each site, all blood samples were tested using the First Response Malaria Ag PLDH/HRP2 Combo rapid test kit (Premier Medical Corporation Private Limited, Gujarat, India) to diagnose malaria. As recommended by the manufacturer, 5 μL of whole blood was transferred into the sample collection well, and about 60 μL of assay buffer was dropped into the assay buffer well. The result was read 20 minutes after the specimen and buffer were added to the test device. A sample was interpreted as positive when two color bands appeared at Control line “C” and the test line “P.f” or “PAN.” The kit has a sensitivity and specificity of 100% and 98.1%, respectively (as stated in the packet insert). Sera, whole blood and BACTEC samples were then transferred to the Naval Medical Research Unit-3 (NAMRU-3) laboratory at Noguchi Memorial Institute for Medical Research located in Accra, Ghana, for bacterial culture, serology, or polymerase chain reaction (PCR) (Figure 1). Follow-up blood samples were obtained 2 to 6 weeks after the primary sample was acquired for serological analysis.

### Serology.

An ELISA was performed using commercially available kits^12^^,^^13^ as follows: The Human *Leptospira* Antibody (IgM and IgG) ELISA kit (Cusabio, Wuhan, China) was used for the qualitative determination of human *Leptospira* antibody concentrations in serum. All reagents in the kit and frozen serum samples were brought to room temperature and concentrated wash buffer (20×) was diluted to 1×. The serum samples were first diluted with the sample diluent and absorbent as instructed by manufacturers before they were added into the microtube strips (eight strips), which were pre-coated with *Leptospira* antigens. Sample dilution preparation for the IgM and IgG ELISA was done by making a 40-fold dilution with the sample diluent. This was achieved by adding 10 μL of sample in 390 μL of sample diluent. For IgM, 100 μL of diluted sample was added to 40 μL of sample absorbent to complete the sample dilution process. All assays were run with positive, negative, and reference (only for IgM) sera, which were provided in the kit. The controls and each sample were read using a microplate reader (BioTek EL 808 microplate reader, Winooski, Vermont, USA) at a wavelength of 450 nm. As recommended by manufacturers, to calculate the human *Leptospira* antibody (IgM and IgG), the optical density (OD) value of the samples was compared with that of the controls. An OD value less than 0.2 was considered negative, whereas an OD value greater than or equal to 0.5 was considered positive. Samples were retested when their OD value was between 0.2 and 0.5. The assay has a high sensitivity and excellent specificity for the detection of human *Leptospira* antibodies (IgM and IgG).

The qualitative measurement of IgM and IgG antibodies against *Coxiella burnetii* (Q fever) in the serum was conducted using the Abcam (Cambridge, United Kingdom) Anti–*Coxiella burnetii* Phase 2 IgM and IgG ELISA kit. Before the reagents and serum samples were used for the assay, they were equilibrated to room temperature. Sample preparation involved making a 1:100 dilution in provided sample dilution buffer, and concentrated wash buffer (20×) was diluted to 1×. After sample dilution was completed, 100 μL of the diluted sample was added to the microtube plate pre-coated with Anti–*Coxiella burnetii* Phase 2 IgM/IgG. *Coxiella burnetii* IgM and IgG controls (positive, negative, blank, and cutoff) were included in duplicate for each assay performed to determine the test result. Downstream analysis was performed according to the manufacturer’s instructions. The controls and each sample were read using a microplate reader (BioTek EL 808 microplate reader) at the wavelength of 450 nm. As recommended by the manufacturer, certain criteria needed to be met for a test to be considered valid. A blank had to be < 0.100, negative control was < 0.200 and less than cutoff, cutoff was control 0.150–1.300, and positive control was greater than cutoff control. To estimate the optical density (OD) value of each sample was first calculated and used to calculate the result of each sample. To calculate the result in a standard unit, we used the following equation:

$$\text{Standard unit}=\frac{\text{Patient serum }OD\text{ value}\times 10}{\text{mean cutoff }OD\text{ value}}$$

A calculated standard unit of < 9 was considered negative and > 11 was considered positive. The kit has a sensitivity and specificity of > 90.

### Bacterial culture.

BACTEC bottles containing blood specimen were incubated overnight for 18 to 24 hours at 35–37°C. The cultured blood sample was collected aseptically by disinfecting the surface of the BACTEC bottle with 70% alcohol prep-pad and using a sterile 1-mL syringe with a thin needle and dropping it on the surface of blood, chocolate, and MacConkey agar and allowing it to dry. The plates were then streaked using a sterile 1-μL inoculating loop and incubated at 35–37°C for 18 to 24 hours. Grown bacterial isolates were identified by colony morphology, Gram staining, biochemical tests (catalase, oxidase, and coagulase), and Analytical Profile index (API 20E from bioMerieux, Inc., Marcy-l’Étoile, France) based on the manufacturer’s instructions and standard procedures.^14^ Blood culture bottles with no bacterial growth after 7 days were subcultured on blood, chocolate, and MacConkey agar (to aid the growth of slow-growing bacteria) before being reported as negative. Leptospirosis culture was performed by inoculating two to three drops of blood in EMJH medium and kept at 37°C in the dark.

### DNA extraction.

The DNA was isolated from blood embedded on a Whatman protein saver card (GE Healthcare Ltd, Chicago. Illinois) using the (Qiagen, Hilden, Germany) blood DNA mini kit following manufacturer’s instructions.

### Nucleic acid amplification.

Real-time PCR targeting the IS*1111a* region of *C. burnetii* was used to detect *C. burnetii* in collected samples.^15^ Lig1/Lig2 primers were used to amplify the conserved amino terminus region of the *ligA* and *B* genes of *Leptospira*. Conventional PCR (Applied Biosystems Machine 9700, Norwalk, CT) was performed using the PCR core kit (New England Biolab, Ipswich, MA) and primers as described by Palaniappan et al.^16^

### Statistical methods.

All statistical analyses were conducted using R 3.3.1 software package. The χ^2^ and Fisher’s exact tests were used to determine significant associations between the presenting symptoms and laboratory test results (ELISA and culture) stratified by malaria status.

Multiple correspondence analysis (MCA) was used to identify data patterns and associations between variables to provide a graphical visualization between active variables (symptoms and sociodemographic characteristics) and supplementary variables (infection status). The infection status consisted of three levels, namely bacterial infection, malaria, and coinfection (bacterial and malaria infections), which were used as the dependent variables. The number of dimensions maintained in the final model was determined using the scree plot.

## RESULTS

A total of 399 participants were enrolled; 222 (55.6%) were male and 177 (44.4%) were female (Supplemental Table 1). Less than half of the patients (48.2%) were younger than 18 years, and 29.8% were younger than 5 years (Supplemental Table 1). In addition to fever, other reported symptoms are listed in Supplemental Table 1, with the most common being headache (319, 79.9%), chills (313, 78.5%), joint pain (166, 41.6%), and muscle pain (151, 37.8%). The commonest clinical diagnoses were malaria (283, 70.9%) and respiratory tract infection (65, 16.3%).

**Figure 2.**
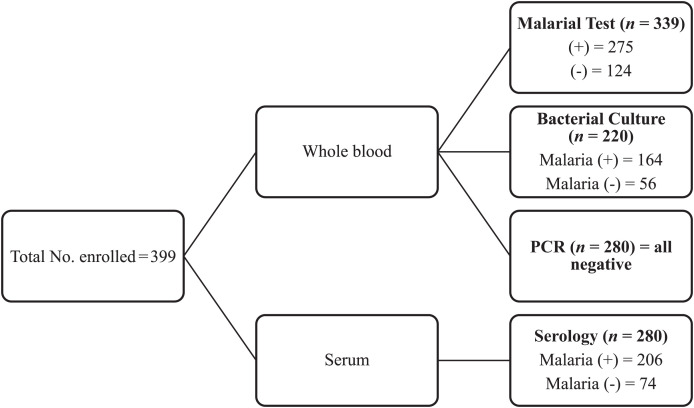
Stratification of the different laboratory assays performed on the various sample types. The chart shows the number of participants enrolled and the different tests performed (malaria, bacterial culture, PCR, and serology). PCR = polymerase chain reaction.

All samples (*N* = 399) were tested for malaria using rapid diagnostic test (RDT) and for leptospirosis and Q fever using PCR. Serological testing was done on 280 samples using serology (participants > 5 years old), and culture was performed on 220 samples (participants > 10 years old because of blood volume limitation) (Figure 1). Malaria RDT was positive in 275/399 participants (68.9%) (Table 1, Figure 2). All PCR results were negative.

**Table 1:** Sociodemographic characteristics, clinical presentation, and coinfection status of malaria-positive patients

Characteristics	Total	Malaria, *N* = 275 (68.9%)							
		Sample tested	Leptospirosis*	*P* value‡	Q fever*	*P* value	Sample tested	Culture positive	*P* value
	*n* (%)	*n* (%)	*n* (%)		*n* (%)		*n* (%)	*n* (%)	
Gender									
Male	158 (567.5)	122 (59.2)	6 (6.0)	0.601	11 (9.0)	0.223	100 (61.0)	5 (0.5)	0.406
Female	117 (42.6)	84 (40.8)	5 (4.9)		13 (15.5)		64 (39.0)	1 (1.6)	
Age (years)†									
< 5	69 (25.1)	–	–	0.052	–	0.401	–	–	–
5–10	42 (15.3)	42 (20.4)	0 (0.0)		8 (19.1)		–	–	–
11–17	25 (9.1)	25 (12.1)	3 (12.0)		2 (8.0)		25 (15.2)	2 (8.0)	0.937
18–29	76 (27.6)	76 (36.9)	3 (4.0)		7 (9.2)		76 (46.3)	3 (3.9)	
30–39	32 (11.6)	32 (15.5)	4 (12.5)		5 (15.6)		32 (19.5)	1 (3.1)	
≥ 40	31 (11.3)	31 (15.0)	1 (3.2)		2 (6.5)		31 (18.9)	0 (0.0)	
Military/Civilian									
Military	43 (15.6)	43 (20.9)	3 (7.0)	0.702	4 (9.3)	0.790	43 (26.2)	2 (4.7)	0.653
Civilian	232 (84.4)	163 (79.1)	8 (4.9)		20 (12.3)		121 (73.8)	4 (3.3)	
Season									
Dry	114 (41.5)	88 (42.7)	6 (6.8)	0.534	7 (8.0)	0.153	76 (46.3)	3 (4.0)	1.000
Rainy	161 (58.6)	118 (57.3)	5 (4.2)		17 (14.4)		88 (53.7)	3 (3.4)	
Symptoms									
Fever									
Current	261 (94.9)	193 (93.7)	10 (5.2)	0.662	23 (11.9)	1.000	152 (92.7)	6 (3.1)	1.000
History of fever	14 (5.1)	13 (6.3)	1 (7.7)		1 (7.7)		12 (7.3)	0 (0.0)	–
Chills									
Yes	224 (81.5)	174 (84.5)	8 (4.6)	0.377	21 (10.9)	1.000	142 (86.6)	6 (4.2)	1.000
No	48 (17.5)	30 (14.6)	3 (10.0)		3 (10.0)		20 (12.2)	0 (0.0)	
Unknown	3 (1.1)	2 (1.0)	0 (0.0)		0 (0.0)		2 (1.2)	0 (0.0)	
Headache									
Yes	228 (82.9)	189 (91.7)	9 (4.8)	0.505	21 (11.1)	0.475	151 (92.1)	5 (3.3)	0.396
No	47 (17.1)	17 (8.3)	2 (11.8)		3 (17.7)		13 (7.9)	1 (7.7)	
Retro-orbital pain									
Yes	54 (19.6)	49 (23.8)	3 (6.1)	0.164	4 (8.2)	0.200	41 (25.0)	2 (4.9)	0.640
No	217 (78.9)	157 (76.2)	8 (5.1)		20 (11.5)		123 (75.0)	4 (3.3)	
Unknown	4 (1.5)	0 (0.0)	–	–	–	–	–	–	–
Conjunctivitis									
Yes	19 (6.9)	16 (7.8)	0 (0.0)	0.738	0 (0.0)	0.286	14 (8.5)	1 (7.1)	0.420
No	252 (91.6)	190 (92.2)	11 (5.8)		24 (11.6)		150 (91.5)	5 (3.3)	
Unknown	4 (1.5)	0 (0.0)	–	–	–	–	–	–	–
Sore throat									
Yes	58 (21.1)	53 (25.7)	0 (0.0)	0.467	5 (9.4)	0.758	39 (23.8)	1 (2.6)	1.000
No	211 (76.7)	152 (73.8)	11 (10.0)		19 (12.5)		124 (7.6)	5 (4.0)	
Unknown	6 (2.2)	1 (0.5)	0 (0.0)	–	0 (0.0)		1 (0.6)	0 (0.0)	
Rash									
Yes	15 (5.4)	11 (5.3)	0 (0.0)	0.329	1 (9.1)	1.000	6 (3.7)	0 (0.0)	1.000
No	259 (94.2)	195 (94.7)	11 (5.6)		23 (11.8)		158 (96.3)	6 (3.1)	
Unknown	1 (0.4)	0 (0.0)							
Muscle pain									
Yes	108 (39.3)	103 (50.0)	2 (1.94)	0.173	10 (9.7)	0.485	93 (56.7)	4 (4.3)	0.712
No	154 (56.0)	101 (49.0)	0 (0.0)		14 (13.9)		70 (42.7)	2 (2.9)	
Unknown	13 (4.7)	2 (1.0)	9 (8.9)	–	0 (0.0)		1 (0.6)	0 (0.0)	
Joint pain									
Yes	126 (45.8)	124 (60.2)	6 (4.8)	0.319	15 (12.1)	1.000	113 (68.9)	4 (3.5)	1.000
No	135 (49.1)	78 (37.9)	5 (6.4)		9 (11.5)		48 (29.3)	2 (4.2)	
Unknown	14 (5.1)	4 (1.9)	0 (0.0)		0 (0.0)		3 (1.8)	0 (0.0)	

Dry season: November to March and rainy season: April to October.

* ELISA (IgM+/IgG−).

† Samples for bacteria culture were not obtained from 42 children from 5 years to 10 years.

‡ *P* value was obtained using χ^2^ or Fisher’s exact test. The *P* value determines the association between the infection status (positive and negative) with the social demographic characteristics and clinical symptoms.

**Figure 3.**
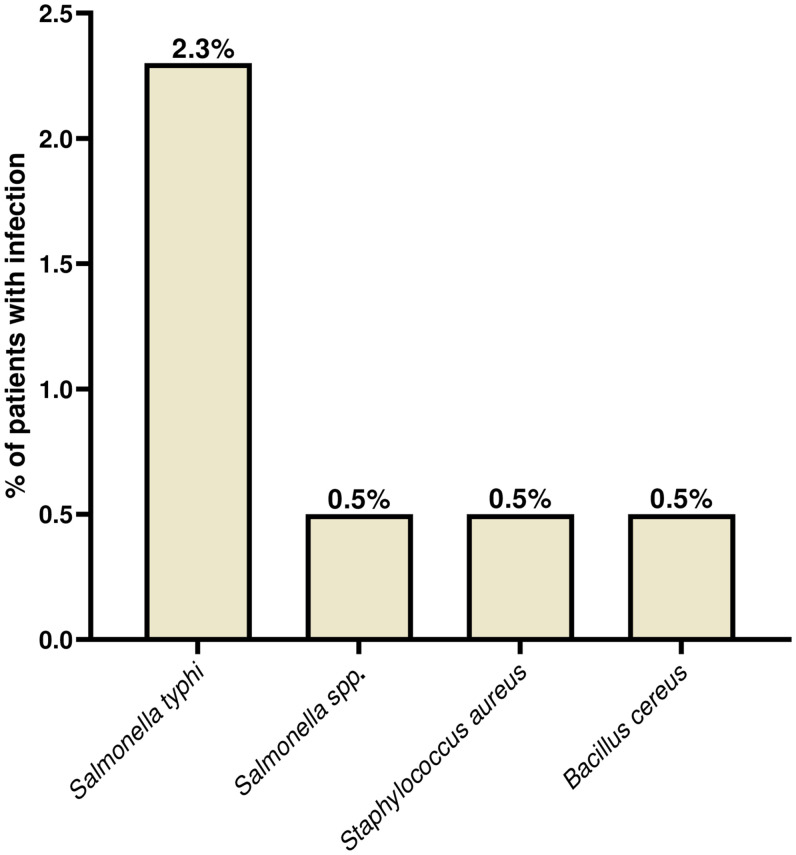
Distribution of bacterial pathogens identified by culture testing is shown for cultured bacteria (*N* = 220). This bar chart shows the proportion of AFI patients confirmed to be exposed to cultured bacterial infection. AFI = acute febrile illness.

Overall, of the 280 serum samples tested, 10.7% and 5.0% were seropositive (IgM+, IgG) for Q fever and leptospirosis, respectively (Figure 3). Of the 220 samples cultured, approximately 8 (4.0%) were positive for a bacterial pathogen including 5 (2.3%) for *S. typhi* and 1 (0.5%) each for *Salmonella* spp., *Staphylococcus aureus*, and *Bacillus cereus* (Table 2, Figure 3). Malaria coinfection occurred with leptospirosis, Q fever, or bacteria in 11/206 (5.3%), 24/206 (11.7%), and 6/164 (3.7%), respectively (Table 3, Figure 2). Among the participants with negative malaria tests, 74 serology and 56 blood culture samples were collected. Of these, 3/74 (4.1%) and 6/74 (8.1%) were seropositive for leptospirosis and Q fever, whereas 2/56 (3.6%) blood cultures were positive (Tables 2 and 3, Figure 2). A total of 50 participants provided paired serum samples, resulting in six and seven acute infections (IgM+, IgG−) of Q fever and leptospirosis, respectively. However, only three and one (IgM+, IgG−) remained for Q fever and leptospirosis, respectively, after follow-up (Table 4). Polymerase chain reaction testing of 280 serum samples did not yield any bacterial DNA.

**Table 2:** Sociodemographic characteristics, clinical presentation of malaria-negative patients with bacterial pathogen

Characteristics	Total	Non-Malaria, *N* = 124 (31.1%)							
		Sample tested	Leptospirosis*	*P* value‡	Q fever*	*P* value	Sample tested	Culture positive	*P* value
	*n* (%)	*n* (%)	*n* (%)		*n* (%)		*n* (%)	*n* (%)	
Gender									
Male	64 (51.6)	42 (56.8)	0 (0.0)	0.077	1 (2.4)	0.079	34 (60.7)	1(2.9)	1.000
Female	60 (48.4)	32 (43.2)	3 (9.4)		5 (15.6)		22 (39.3)	1(4.6)	
Age (years)†									
< 5	50 (40.3)	–	–	–	–	–	–	–	–
5–10	18 (14.5)	18 (24.3)	1 (5.6)	–	4 (22.2)	–	–	–	–
11–17	3 (2.4)	3 (4.1)	0 (0.0)	0.381	0 (0.0)	0.223	3 (5.4)	0 (0.0)	0.630
18–29	30 (24.2)	30 (40.5)	0 (0.0)		1 (3.3)	–	30 (53.6)	2 (6.7)	–
30–39	19 (15.3)	19 (25.7)	2 (10.5)		1 (5.3)		19 (33.9)	0 (0.0)	
≥ 40	4 (3.2)	4 (5.4)	0 (0.0)		0 (0.0)		4 (7.1)	0 (0.0)	
Military/Civilian									
Military	16 (12.9)	16 (21.6)	0 (0.0)	1.000	0 (0.0)	0.329	16 (28.6)	0 (0.0)	1.000
Civilian	108 (87.1)	58 (78.4)	3 (5.2)		6 (10.34)		40 (71.40	2 (5.0)	
Season									
Dry	34 (27.4)	21 (15.5)	1 (4.8)	1.000	0 (0.0)	0.175)	17 (30.4)	1 (5.9)	0.519
Rainy	90 (72.6)	53 (84.5)	2 (3.8)		6 (1.3)		39 (69.6)	1 (2.6)	
Symptoms									
Fever									
Current	116 (93.6)	69 (93.2)	2 (2.9)	0.192	6 (8.7)	1.000	51 (91.1)	2 (3.9)	1.000
History of fever	8 (6.5)	5 (6.7)	1 (20.0)		0 (0.0)		5 (8.9)	0 (0.0)	
Chills									
Yes	89 (71.8)	59 (79.7)	1 (1.7)	0.103	3 (5.1)	0.093	47 (83.9)	1 (2.1)	0.298
No	35 (28.3)	15 (20.3)	2 (13.3)		3 (20.0)		9 (16.1)	1 (11.1)	
Headache									
Yes	91 (73.4)	66 (89.2)	3 (4.6)	1.000	5 (7.6)	0.510	51 (91.1)	0 (0.0)	0.006
No	33 (26.6)	8 (10.8)	0 (0.0)		1 (12.5)		5 (8.9)	2 (40.0)	
Retro-orbital pain									
Yes	21 (16.9)	17 (23.0)	0 (0.0)	1.000	0 (0.0)	0.380	15 (26.8)	0 (0.0)	1.000
No	101 (81.5)	56 (75.7)	3 (5.4)		6 (10.7)		41 (73.2)	2 (3.6)	
Unknown	2 (1.6)	1 (1.4)	0 (0.0)		0 (0.0)		0 (0.0)	0 (0.0)	
Conjunctivitis									
Yes	13 (10.5)	8 (10.8)	0 (0.0)	1.000	0 (0.0)	1.000	7 (12.5)	0 (0.0)	1.000
No	110 (88.7)	65 (87.8)	3 (4.6)		6 (9.2)		48 (85.7)	2 (4.2)	
Unknown	1 (0.8)	1 (1.4)	0 (0.0)	–	0 (0.0)		1 (1.8)	0 (0.0)	
Sore throat									
Yes	32 (25.8)	27 (36.5)	2 (7.4)	0.550	3 (11.1)	0.662	19 (33.9)	1 (5.3)	1.000
No	90 (72.6)	47 (63.5)	1 (2.1)		3 (6.4)		37 (66.1)	1 (2.7)	
Unknown	2 (1.6)	–	–	–	–	–	–	–	–
Rash									
Yes	12 (9.7)	4 (5.4)	0 (0.0)	1.000	0 (0.0)	1.000	4 (7.1)	0 (0.0)	1.000
No	112 (90.3)	70 (94.6)	3 (4.3)		6 (8.6)		52 (92.9)	2 (2.9)	
Muscle pain									
Yes	43 (34.7)	40 (54.1)	1 (2.5)	0.591	1 (2.5)	0.088	36 (64.3)	1 (2.8)	1.000
No	79 (63.7)	34 (45.9)	2 (5.9)		5 (14.7)		20 (35.7)	1 (5.0)	
Unknown	2 (1.6)	0 (0.0)	–	–	–	–	–	–	–
Joint pain									
Yes	40 (32.3)	38 (51.4)	1 (2.6)	0.610	1 (2.6)	0.103	33 (58.9)	1 (3.0)	1.000
No	82 (66.1)	36 (48.6)	2 (5.6)		5 (13.9)		23 (41.1)	1 (4.4)	
Unknown	2 (1.6)	0 (0.0)	–	–	–	–	–	–	–

Dry season: November to March and rainy season: April to October.

* ELISA (IgM+/IgG−).

† Samples for bacterial culture were not obtained from 18 children from 5 to 10 years of age.

‡ *P* value was obtained using χ^2^ or Fisher’s exact test. The *P* value determines the association between the infection status (positive and negative) with the social demographic characteristics and clinical symptoms.

**Table 3:** Comparison of coinfections in the malaria positives and negatives with bacterial infection

Bacterial infection	Total *n* (%)	Malaria		
		Positive	Negative	*P* value
		*n* (%)	*n* (%)	
Leptospirosis	–	–	–	0.663
Positive	14 (5.0)	11 (5.3)	3 (4.1)	–
Negative	266 (95.0)	195 (94.7)	71 (95.5)	–
Q fever	–	–	–	0.398
Positive	30 (10.7)	24 (11.7)	6 (8.1)	–
Negative	250 (89.3)	182 (88.4)	68 (91.9)	–
Culture*	–	–	–	0.976
Positive	8 (3.6)	6 (3.7)	2 (3.6)	–
Negative	212 (96.4)	158 (96.3)	54 (96.4)	–

* Sixty participants from 5 to 10 years of age did not provide samples for bacterial culture.

**Table 4:** *Coxiella burnetii* and *Leptospira* spp. detection in paired sera

*C. burnetii*			*Leptospira* spp.		
Antibodies	Initial (*n*)	Follow-up	Antibodies	Initial (*n*)	Follow-up (*n*)
IgM(+)IgG(−)	3	3	IgM(−)IgG(−)	5	5
IgM(+)IgG(−)	3	3	IgM(+)IgG(−)	1	1
IgM(+)IgG(−)	0	0	IgM(−)IgG(+)	1	1
IgM(−)IgG(−)	9	9	IgM(+)IgG(−)	0	0
IgM(−)IgG(+)	0	0	IgM(−)IgG(−)	1	1
IgM(−)IgG(−)	35	35	IgM(−)IgG(−)	42	42

The first three MCA dimensions explain approximately 35% of the variability among febrile patients with an infection (Supplemental Table 2, Supplemental Figure 1). Variables defining the first dimension were mainly associated with or without sore throat and joint and muscle pain. The most discriminant variables for dimension 2 were age, with or without muscle and joint pain. Variables that were mainly associated with dimension 3 were age, with or without headache, retro-orbital pain, and conjunctivitis.

**Figure 4.**
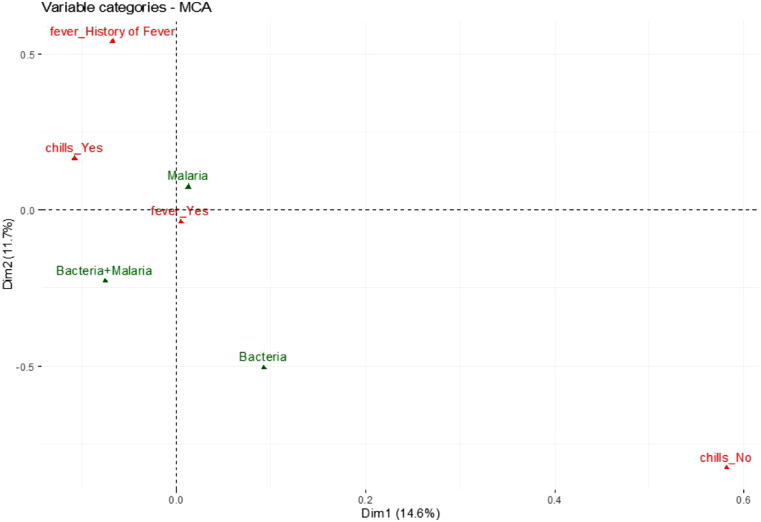
Multiple corresponding analysis (MCA) plot in R of dimensions 1 and 2 (Dim 1, Dim 2) with the active variables (yes/no) fever and chills (red) vs. infection as a supplementary variable (green). The plot represents a two-dimensional graph (dimensions 1 and 2) that indicates the significant impact of the active and supplementary variables on the dimensions.

**Figure 5.**
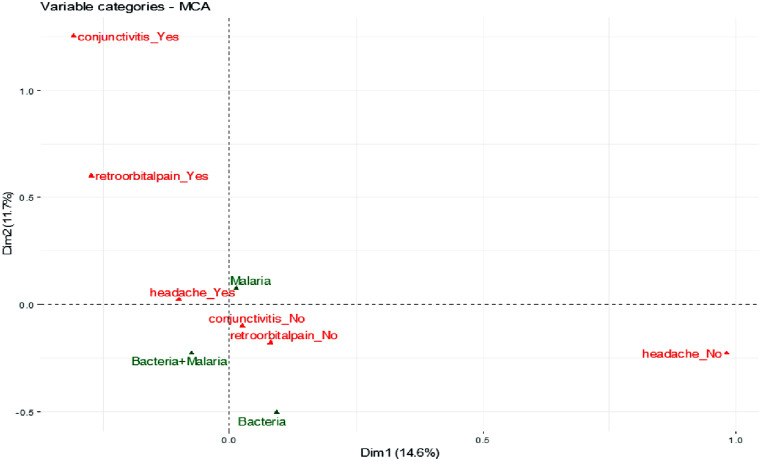
Multiple corresponding analysis (MCA) plot in R of dimensions 1 and 2 (Dim 1, Dim 2) with the active variables (yes/no) conjunctivitis, retro-orbital pain, and headache (red) vs. infection as a supplementary variable (green). The plot represents a two-dimensional graph (dimensions 1 and 2) that indicates the significant impact of the active and supplementary variables on the dimensions.

**Figure 6.**
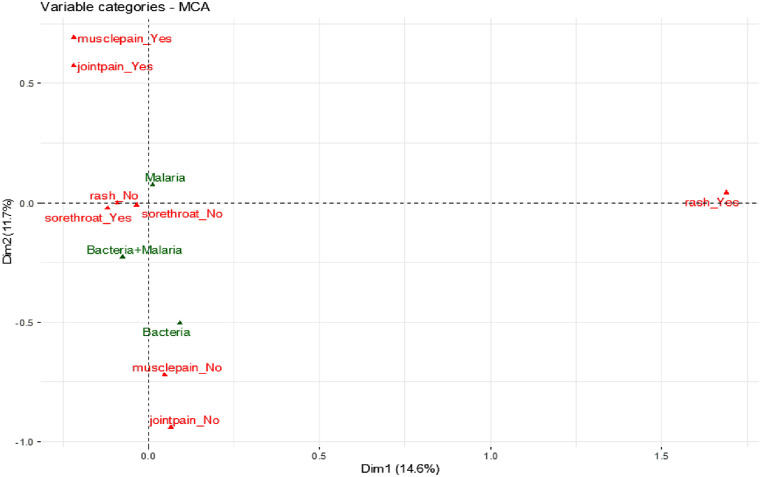
Multiple corresponding analysis (MCA) plot in R of dimensions 1 and 2 with the active variables (yes/no) rashes, muscle pain, and joint pain (red) vs. infection as a supplementary variable (green). The plot represents a two-dimensional graph (dimensions 1 and 2) that indicates the significant impact of the active and supplementary variables on the dimensions.

The clinical presentation of fever without any other symptoms did not correlate specifically to any of the infections based upon its close proximity to the origin in the MCA analysis, suggesting it cannot be used to distinguish between malaria, bacterial infections, and coinfections (Figure 4), although coinfection was associated with headaches and sore throat. The presence of retro-orbital pain, conjunctivitis, joint pain, and muscle pain correlated to malaria alone rather than to malaria with bacterial coinfection (Figures 5 and 6). The MCA analysis confirmed data from Table 2 that bacterial infection did not correlate with headaches (Figure 5). In addition, bacterial infection correlated with rashes but not with retro-orbital, muscle, or joint pain, conjunctivitis, and chills (Figures 5 and 6). The risk of a febrile patient having an infection was not strongly correlated with age, sex, chills or fever (Supplemental Table 2) as compared to sore throat, muscle pain, and joint pain.

## DISCUSSION

Although the WHO reports a global decline in malaria since 2010, it remains a significant problem in sub-Saharan Africa. Despite the positive impact of presidents malaria initiative (PMI) and other malaria initiatives, Ghana—a country with one of the highest burdens of malaria—reported an increased incidence of malaria cases in 2018.^17^ Therefore, it was not surprising that malaria was highly prevalent (68.9%) in this study of AFI patients. Malaria parasite and bacterial bloodstream coinfections were common among patients with severe febrile illness in a review of low- and middle-income countries by Prasad et al.^18^ In Ghana, malaria coinfections with bacteremia have been reported in children,^7^^,^^19^ but very little information exists about malaria with other zoonotic bacteria. Our results showed 11.7% Q fever, 5.3% leptospirosis, and 3.7% bacterial pathogen coinfection rates with malaria. This study revealed the presence of some bacterial zoonotic and bloodstream infections as contributors to AFIs in our hospitals. A systematic review of diverse zoonoses causing febrile illness in malaria-endemic regions revealed that the majority of the contributing causes of fever were due to bacterial infections.^5^ We also recorded 4.1% leptospirosis, 8.1% *C. burnetii*, and 3.6% bacteremia in patients who were malaria negative, identifying these pathogens as contributing to the burden of nonspecific febrile cases in Ghana.

The overall seropositivity against Q fever antibody was 10.7%, similar to 9.6% IgM seropositivity reported in a febrile study in Mali.^20^ Other reports of seropositivity of < 8% have been recorded in Chad, the Ivory Coast, and Tanzania.^21^ In Ghana, Q fever infection in febrile patients was scarcely detected with some reports in children under 5 years old.^8^ The 5% seropositivity of leptospirosis observed in this study was lower than the 11.5% reported in a study among febrile patients in Mozambique.^22^ Our result is also in the range of 0–33% reported in a review of leptospirosis in sub-Saharan Africa by de Vries et al.^23^ This study was conducted in two urban cities in Ghana (Accra and Sekondi-Takoradi); hence, our outcome could justify the observation that leptospirosis, which is a well-known rural disease, could also occur in urban settings, which often comprise poor urban slums,^24^ but at slightly lower rates.

Seroconversion of the initial and follow-up serum samples was seen in both Q fever and leptospirosis infections. Nine serum samples that were initially negative for acute testing were found to be positive (IgM + IgG−) during follow-up testing for Q fever, adding up to the three serum samples that were positive in the initial testing. Likewise, for leptospirosis, seven serum samples were positive (IgM+ IgG−) for the initial testing, one serum sample seroconverted from IgM+ IgG− to IgM− IgG+, with one serum sample remaining IgM− IgG+, confirming that IgG can remain in the bloodstream for a long time. This finding confirms the need for paired sera testing because it is known to take about 7 to 15 days or more after the onset of illness for seroconversion^25^ Immunoglobulin M antibody for these infections takes a few weeks to develop during acute infection.

*Coxiella burnetii* and *Leptospira* DNA were not detected by PCR; however, antibodies were detected by ELISA. Even though serology alone may not prove infection with Q fever and leptospirosis, it has been documented that as antibodies build, nucleic acid detection decreases to levels below the PCR detection limit.^26^ This may explain some of the discordant results between nucleic acid amplification and ELISA results.

Bacterial bloodstream infection and bacterial zoonoses have been reported as major causes of fever.^5^ For 220 samples cultured, approximately 2.3% of *S. typhi* and 0.5% of *Salmonella* spp., *S. aureus,* and *B. cereus* each were identified (Figure 3). *Salmonella typhi* is known as one of the more frequently isolated blood-borne pathogens in Ghana.^27^ Most studies have shown high seroprevalence of enteric fever using the Widal tests, which have a high cross-reactivity rate.^28^ The low prevalence seen in this study is similar to that of some studies reported in Ethiopia,^28^ Nigeria,^29^ and Ghana.^30^ This could be attributed to irregular shedding of the bacteria or to the majority of patients being from urban areas with improved access to clean water and healthcare. Globally *S. aureus* is known to cause bacteremia, sepsis, and skin infections^31^ in hospital or community-acquired settings.^32^ We found a relatively low percentage (0.5%) of *S. aureus* isolates in this study, similar to a Ghanaian study that reported an isolation rate of 0.6% among febrile patients in rural hospitals.^33^ *Bacillus cereus* is commonly associated with gastrointestinal infection; however, it has increasingly been reported as one of the potentially important pathogens causing nosocomial bloodstream infections.^34^^,^^35^ Our study shows culture-proven bacterial coinfections occur, but are rare.

None of the presenting signs and symptoms were significantly associated with a particular clinical disease with use of Fisher’s exact or χ^2^ test. This demonstrates the nonspecific presentation of malaria and its overlap with the pathogens responsible for AFI.^18^^,^^36^^,^^37^ Therefore, signs and symptoms alone are unlikely to discern the cause of AFIs based solely on clinical presentation, highlighting the need for enhanced diagnostic assays.^38^^,^^39^ However, the MCA helped to visualize probable associations among symptoms, single pathogen infections, and coinfections. In our study, we found that febrile patients presenting with retro-orbital pain, conjunctivitis, and joint and muscle pain were more likely to have a malaria infection alone as opposed to malaria with a bacterial coinfection. The MCA proved productive, showing possible links between symptoms and infection. This could be considered an important additional analytical tool when associations of categorical variables are analyzed.

Our findings suggest that patients 5 years of age and older irrespective of having muscle and joint pain correlated with malaria infection rather than malarial-bacterial coinfection. Njeru and others^40^ mentioned attempts to predict Q fever, malaria, and typhoid infections based on clinical signs and symptoms modeling. However, like our study, most symptoms were not suggestive of a particular infectious etiology and therefore could not be used to increase the pretest probability of any particular infection. This highlights the challenges of identifying the causes of AFIs solely based on clinical presentation in austere setting. It also emphasizes the importance of improving diagnostics to better characterize the distribution of AFI-causing pathogens. In this study, a febrile person’s age and gender, together with experiencing chills or fever, did not strongly correlate with the infections we studied (Supplemental Table 2), unlike a study in Nigeria that reported the highest prevalence of *Salmonella* infections was found in young adults.^29^

Our limited findings in this austere environment demonstrate the need for more comprehensive diagnostic modalities—ones that complement the findings of the clinical exam—to better understand the multiple etiologies contributing to AFIs in tropical environments such as Ghana and West Africa. Continuous febrile illness surveillance can help inform local epidemiology and febrile illness management and will be essential for the detection of disease outbreaks.^41^

## LIMITATIONS

This study was subject to limitations that are associated with using commercial ELISA kits and malaria RDT kits. These are prone to cross-reactivity among pathogens. Acquiring follow-up samples for paired testing was a challenge, as mentioned in other febrile illness research.^7^^,^^36^ Another important limitation to our study was the low rate of follow-up, which was mostly attributed to early patient recovery, inability to take time off work, and other unforeseen circumstances. It is understood that children younger than 5 years may be more prone to malarial-bacterial coinfections; however, because of ethical concerns, we could not draw large volumes of blood from this group. Thus, not all tests were performed on their specimens. Another limitation was the site-by-site variation in sample size due to the smaller number of participants enrolled from Accra (*n* = 71) compared with Takoradi (*n* = 328).

## CONCLUSIONS

Although positive rapid diagnostic tests for malaria are common among Ghanaians from urban settings presenting with fever, about 10% of participants have evidence suggesting at least one other bacterial cause of AFI. Therefore, additional etiologies for fever should be considered when patients present with AFI, even in the setting of a positive malaria test. In general, clinical signs and symptoms were not significantly associated with the presence of coinfections, but retro-orbital pain, conjunctivitis, joint pain, and muscle pain were more likely among those who had a positive malaria rapid diagnostic test and lacked evidence of coinfections.

This study highlights that bloodstream infections, leptospirosis, and Q fever could contribute to AFIs in Ghana. We report that coinfections with these pathogens and malaria could range from 3–11% among febrile patients in Ghana. We also found that fever without any other symptoms could not be used to accurately distinguish between malaria, bacterial infections, and coinfections. Symptoms associated with malaria included retro-orbital pain, conjunctivitis, and joint and muscle pain, whereas malaria coinfection with Q fever or leptospirosis was associated with headaches and sore throat. Laboratory-confirmed bacterial diagnosis of AFIs should be improved because clinical presentation alone is too similar to malaria to discriminate the presence of other coinfections.

## Supplemental Materials


Supplemental materials
