# Supplementary material for: Coinfection of Malaria and Bacterial Pathogens among Acute Febrile Patients in Selected Clinics in Ghana
Source: Am J Trop Med Hyg. 2023 Sep 25;109(5):1036–46. doi: 10.4269/ajtmh.23-0099 (PMC10622490; doi:10.4269/ajtmh.23-0099)
Supplement: Supplementary file 1 [file tpmd230099.SD1.pdf]

SUPPLEMENTARY INFORMATION

Table S1: Social demographic characteristics and clinical symptoms of patients and bacterial pathogens identified

Characteristics	Total n (%)	Sample tested n (%)	[b]Leptospirosis n (%)	*p*-value	[b]Q-fever n (%)	*p*-value	Sample tested n (%)	Culture positive n (%)	*p*-value
**Gender**									
Male	222(55.6)	164(58.6)	6(3.7)	0.221	12(7.3)		134(60.9)	6(4.5)	
Female	177(44.4)	116(41.4)	8(6.9)		18(15.5)	0.029	86(39.1)	2(2.3)	0.487
**[a]Age(years)**									
<5.	119(29.8)								
5-10	60(15.0)	60(21.4)	1(1.7)		12(20.0)				
11-17	28(7.0)	28(12.7)	3(10.7)		2(7.1)		28(12.7)	2(7.1)	
18-29	106(26.6)	106(37.9)	3(2.8)	0.047	8(7.6)	0.135	106(48.2)	5(4.7)	
30-39	51(12.8)	51(18.2)	6(11.8)		6(11.8)		51(23.2)	1(2.0)	0.428
40	35(8.8)	35(12.5)	1(2.9)		2(5.7)		35(15.9)	0(0.0)	
**Military/Civilian**									
Military	59(14.8)	59(21.1)	3(5.1)		4(6.7)		59(26.8)	2(3.4)	
Civilian	340(85.2)	221(78.9)	11(5.0)	1.000	26(11.8)	0.348	161(73.2)	6(3.70	1.000
**Season**									
Dry	148(37.1)	109(38.9)	7(6.4)		7(6.4)		93(42.3)	4(4.3)	
Rainy	251(62.9)	171(61.1)	7(4.1)	0.409	23(13.5)	0.075	127(57.7)	4(3.2)	0.724
Symptoms									
**Fever**									
Current	377(94.5)	262(93.6)	12(4.6)	0.283	29(11.1)		203(92.3)	8(3.9)	1.000
History of Fever	22(5.5)	18(6.4)	2(11.1)		1(5.6)	0.711	17(0.07)	0(0.0)	
**Chills**									
Yes	313(78.5)	233(83.2)	9(3.9)	0.159	24(10.3)		189(85.9)	7(3.7)	
No	83(20.8)	45(16.1)	5(11.1)		6(13.3)	0.790	29(13.2)	1(2.2)	1.000
Unknown	3(0.8)	2(0.7)	0(0.0)		0(0.0)		2(0.9)	0(0.0)	
**Headache**									
Yes	319(79.9)	255(91.1)	12(4.7)	0.471	26(10.2)	0.371	202(91.8)	5(2.5)	0.020
No	80(20.1)	25(8.9)	2(8.0)		4(16.0)		18(8.2)	3(16.7)	

[a]Samples for bacteria culture was not obtain from 60 children from 5 years to 10 years.

[b]ELISA (IgM+/IgG-)

**p- value* was obtained using Chi-square or Fisher's exact test were necessary: The *p*-value determines the association between the infection status (positive and negative) with the social demographic characteristics and clinical symptoms

Dry Season: November to March and Rainy Season: April to October

Table S1 Continued: Social demographic characteristics and clinical symptoms of patients and bacterial pathogens identified

Characteristics	Total n (%)	Sample tested n (%)	[b]Leptospirosis n (%)	*p*-value	[b]Q-fever n (%)	*p*-value	Sample tested n (%)	Culture positive n (%)	*p*-value
**Retro-orbital pain**									
Yes	75(18.8)	66(23.6)	3(4.6)		4(6.1)		56(25.5)	2(3.6)	
No	318(79.7)	213(76.1)	11(5.6)	1.000	26(12.2)	0.440	164(74.5)	6(3.7)	1.000
Unknown	6(1.5)	1(0.4)	0(0.0)		0(0.0)		0(0.0)	0(0.0)	
**Conjunctivitis**									
Yes	32(8.0)	24(8.6)	0(0.0)	0.637	0(0.0)		21(9.5)	1(4.8)	
No	362(90.7)	255(91.1)	14(5.5)		30(11.8)	0.186	198(90.0)	7(3.5)	0.576
Unknown	5(1.3)	1(0.4)	0(0.0)		0(0.0)		1(0.5)	0(0.0)	
**Sore throat**									
Yes	90(22.6)	80(28.6)	2(2.5)		8(10.0)		58(26.4)	2(3.5)	
No	211(76.7)	199(71.7)	12(6.0)	0.395	22(11.6)	1.000	161(73.2)	6(3.7)	1.000
Unknown	8(2.0)	1(0.4)	0(0.0)		0(0.0)		1(0.5)	0(0.0)	
**Rash**									
Yes	27(6.8)	15(5.4)	0(0.0)	1.000	1(6.7)		10(4.5)	0(0.0)	1.000
No	371(93.0)	265(94.6)	14(5.3)		29(10.9)	1.000	210(95.5)	8(3.8)	
Unknown	1(0.3)	0(0.0)							
**Muscle Pain**									
Yes	151(37.8)	143(51.1)	3(2.1)		11(7.7)		129(58.6)	5(3.9)	
No	233(58.4)	135(48.2)	11(8.2)	0.065	19(14.1)	0.270	90(40.9)	3(3.3)	1.000
Unknown	15(3.8)	2(0.7)	0(0.0)		0(0.0)		1(0.5)	0(0.0)	
**Joint Pain**									
Yes	166(41.6)	162(57.9)	7(4.3)		16(9.9)		146(66.4)	5(3.4)	
No	217(54.4)	114(40.7)	7(6.1)	0.659	14(12.3)	0.721	71(32.3)	3(4.2)	1.000
Unknown	16(4.0)	4(1.4)	0(0.0)		0(0.0)		3((1.4)	0(0.0)	

[b]ELISA (IgM+/IgG-)

**p- value* was obtained using Chi-square or Fisher's exact test were necessary: The *p*-value determines the association between the infection status (positive and negative) with the social demographic characteristics and clinical symptoms.

Table S2: Significance test result of contributing factors in the top three dimensions

	Dimension 1		Dimension 2		Dimension 3	
	R^2	*p*-value	R^2	*p*-value	R^2	*p*-value
Age	-		0.36	<0.001	0.2	<0.001
Sex	-		0.09	<0.001	-	
Fever	-		0.02	<0.001	0.18	<0.001
Chills	0.06	<0.001	0.14	<0.001	0.08	<0.001
Headache	0.09	<0.001	-	<0.001	0.27	<0.001
Retro-orbital Pain	0.02	<0.001	0.11	<0.001	0.31	<0.001
Conjunctivitis	-		0.12	<0.001	0.22	<0.001
Sore throat	0.7	<0.001	0.03	<0.001	0.04	<0.001
Rashes	0.15	<0.001	-	<0.001	-	
Muscle pain	0.79	<0.001	0.53	<0.001	0.03	<0.001
Joint pain	0.6	<0.001	0.58	<0.001	0.09	<0.001
% Variance	14.6		11.7		8.9	

R^2 is the coefficient of determination, which measures the percentage of variability within the dimension that are explained by the predictors.

p-value measures the association between the predictors and the dimension

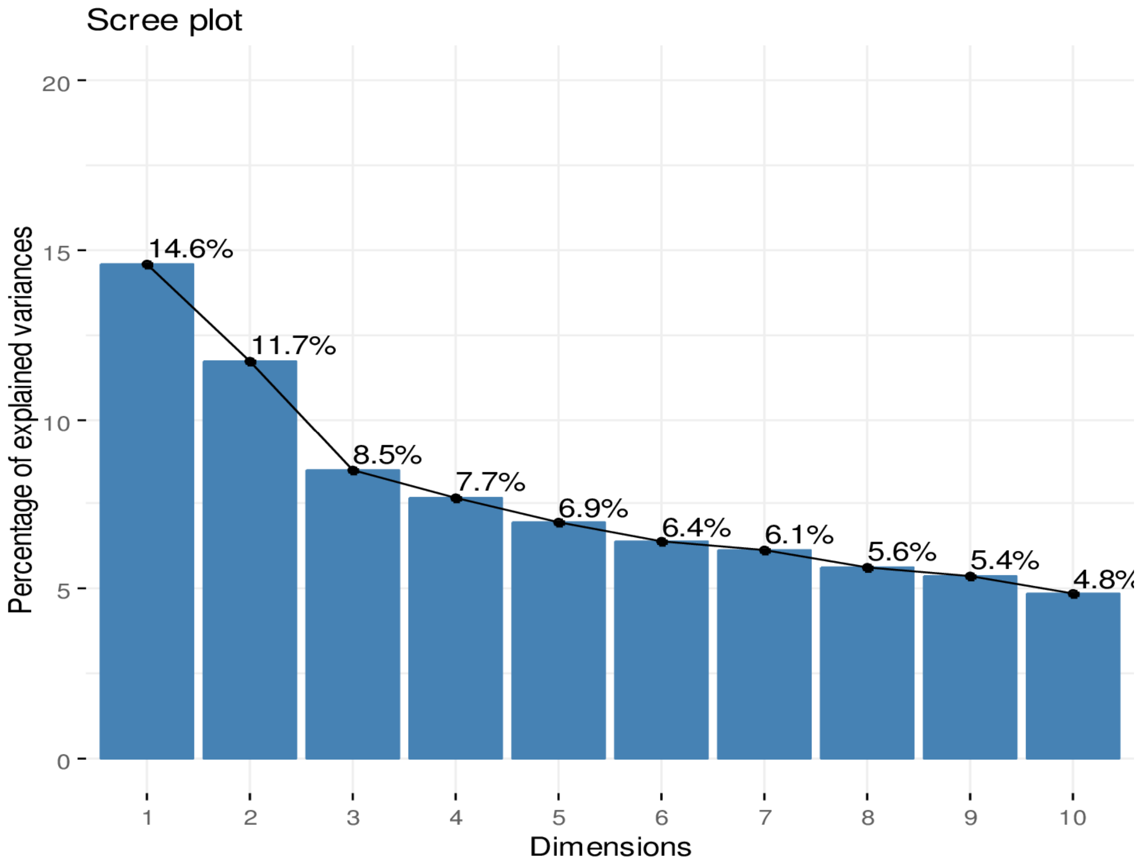

S1 Figure: Determining the optimum number of dimensions using a scree plot. The plot shows the explained variation and the dimension which indicates that dimensions 1, 2 and 3 are enough to describe the data.

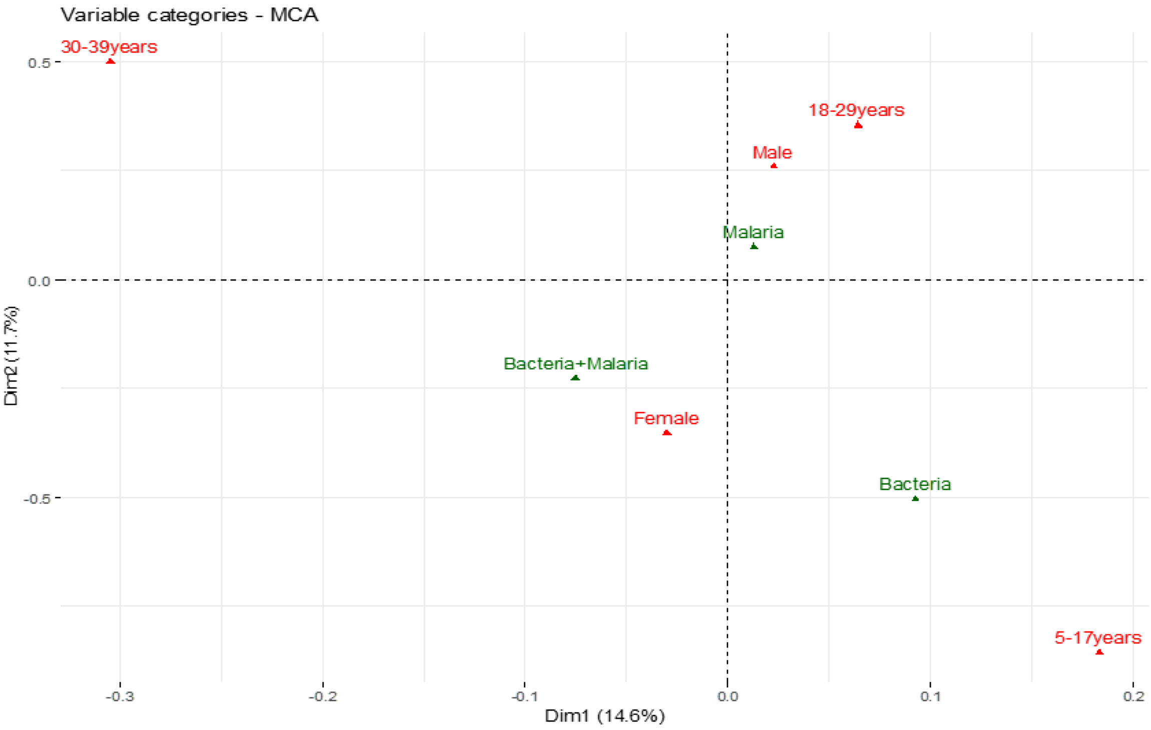

S2 Figure: Multiple corresponding analysis (MCA) plot in R of dimensions 1 and 2 with the active variables age, and gender (red) versus infection as a supplementary variable (green). The plot represents a two-dimensional graph (dimensions 1 and 2) which indicates the significant impact of the active and supplementary variables on the dimensions.
